# Zero human deaths from dog-mediated rabies by 2030: perspectives from quantitative and mathematical modelling

**DOI:** 10.12688/gatesopenres.13074.2

**Published:** 2020-03-04

**Authors:** WHO Rabies Modelling Consortium

**Keywords:** canine rabies, WHO guidelines, post-exposure prophylaxis, validation, verification, mass dog vaccination, zoonosis, surveillance, integrated bite case management

## Abstract

Dog-mediated rabies continues to kill tens of thousands of people every year in low- and middle-income countries despite being an entirely vaccine-preventable disease. WHO and partners have launched a global campaign to reach zero human deaths from dog-mediated rabies by 2030. The primary tools for reaching this target are mass dog vaccination to interrupt transmission in domestic dog populations that maintain infection, appropriate post-exposure prophylaxis (PEP) for rabies-exposed persons to prevent the fatal onset of disease, together with education to support their effective uptake. Models have been developed to assess the feasibility, impact and cost-effectiveness of these measures. From these models, we argue that the 2030 target of zero human rabies deaths is achievable, but will require concerted effort, engagement and investment. A proposed Gavi investment in human rabies vaccines has potential to drive progress towards the 2030 target; however, concomitant investment is needed to scale up mass dog vaccination or this target will be missed. Predicted economic benefits of mass dog vaccination vary according to national PEP provisioning and healthcare access. Integrated Bite Case Management can enhance surveillance and rationalize PEP use, but needs adapting to and integrating within local health systems and international reporting systems to improve PEP accountability, monitor impacts and support verification of disease freedom. Modelling approaches need refining to project realistic and geographically specific timelines for achieving targets. Model iterations informed by data on the implementation of interventions can be used to evaluate progress and guide future strategies. Critically such models are needed to advocate for investment, since the greatest risk to the ‘Zero by 30’ strategy is the limited long-term cross-sectoral or targeted financing to support countries to deliver and sustain mass dog vaccination.

## Disclaimer

The views expressed in this article are those of the author(s). The opinions expressed herein are those of the authors and do not necessarily reflect the views of the World Health Organisation. Publication in Gates Open Research does not imply endorsement by the Gates Foundation.

## Background

In over 120 countries around the world, rabies presents a significant threat to human lives and a considerable public health burden. Around 60,000 people die from rabies annually, whilst tens of millions receive costly post-exposure prophylaxis (PEP) ^1^. The vast majority of these human rabies cases (>99%) are contracted through the bite of a rabid dog ^2^. Although fatal following the onset of clinical disease, rabies is entirely preventable. Administration of PEP to rabies exposed persons according to WHO guidelines prevents disease progression and death ^3^. However, PEP does not reduce transmission in source domestic dog populations. Mass dog vaccination has been the foundation for the successful elimination of rabies from dogs in North America, Western Europe, Japan and much of Central and South America ^2^. In contrast, mass dog vaccination has barely started in most low- and middle-income countries (LMICs) in Africa and Asia, where the disease remains widespread.

Within the context of neglected tropical diseases (NTDs), zoonoses like rabies have been especially neglected. Rabies was not amongst the diseases prioritized in the 2012 London Declaration, endorsed by international, non-government, government and industry partners that committed over US$785 million to the control or elimination of 10 NTDs by 2020. Investment in the 2012 roadmap overlooked zoonoses requiring control of disease in animal or vector reservoirs ^4^. However, rabies was listed amongst five Neglected Zoonotic Diseases (NZDs) within the 66th World Health Assembly resolution on NTDs in 2013 ^5^. The lack of progress on rabies control in Africa and Asia reflects the omission from high-level political advocacy ^6^, and the consequences of minimal investment. A One Health approach is increasingly advocated, recognizing the interactions between human and animal health and the required intersectoral collaboration at local, national and international levels. But concomitant investment in veterinary public health to deal with endemic zoonoses has been limited. Without financing mechanisms, veterinary services in most LMICs will remain focused on livestock diseases considered valuable for trade and will lack the resources needed for mass dog vaccination.

WHO and partners recently launched a global campaign to address the neglect of rabies, with the aim of achieving zero human deaths from dog-mediated rabies by 2030 ^7^. Some regions already had targets to interrupt transmission in dogs or reach zero human deaths. The Americas have progressed remarkably: human rabies deaths in the region have declined by >99% from endemic levels, many countries have not reported human deaths from dog-mediated rabies in over a decade (only three countries reported deaths in 2016 ^8^), and have ostensibly eliminated transmission, with mass dog vaccination discontinued from large areas. Although the Americas had to reset their target dates and are still to verify freedom from dog-mediated rabies, their successes demonstrate what is possible through coordinated mass dog vaccination. The Southeast Asia and Western Pacific region’s targets for zero deaths from 2020 ^9^ have both been reset following limited progress. The Pan-African Rabies Control Network (PARACON) and Association of Southeast Asian Nations (ASEAN) united regional groups across Africa and Southeast Asia, respectively, and are involved in pushing the rabies elimination agenda. However, with a few exceptions ^10^, countries on both continents have yet to demonstrate significant case reductions. Development of a new NTD roadmap for 2030 that includes rabies provides an opportunity to build on successes and reflect on failures to constructively identify obstacles and overcome them.

For rabies, the new 2030 NTD roadmap, for the first time, identifies time-bound targets for countries to achieve the long-term goal of zero human rabies deaths, intermediate milestones of 50% mortality reduction and operational prerequisites of 70% mass dog vaccination coverage in high-risk areas ^11^. Modelling contributed to policy development in the build up to and subsequent launch of the ‘Zero by 30’ campaign. In this letter, we describe insights from modelling that can inform progress towards the 2030 NTD roadmap and lay out challenges to meeting these targets.

## Insights from modelling supporting policy development

The historic lack of investment in NZDs has previously been attributed to an absence of estimates of their burden on society to motivate policy ^12– 14^. Human and animal rabies is vastly under-reported in most endemic regions due to a lack of surveillance and reporting capacity, contributing to this cycle of neglect ^15, 16^. With this in mind, the Global Alliance for Rabies Control commissioned a study to estimate the global burden of dog-mediated rabies. Building on decision tree models ^17, 18^ and using updated country data, burden estimates were generated that have been widely cited in advocacy ^1, 19^.

Gavi, the Vaccine Alliance, has considered investing in rabies as part of its mission to increase the equitable use of vaccines for children in the world’s poorest countries. The Gavi Learning Agenda on rabies, the outcome of Gavi’s 2013 Vaccine Investment Strategy decision, complemented key policy processes: a Strategic Advisory Group of Experts (SAGE) Working Group reviewed evidence on rabies vaccines and immunoglobulins ^20^ (July 2016–April 2018) and a review of models on the impact and cost-effectiveness of rabies prevention strategies was undertaken through the Immunization and Vaccines related Implementation Research Advisory Committee (IVIR-AC) in 2017. Both SAGE and IVIR-AC models highlighted that one strategy under consideration, rabies pre-exposure prophylaxis within a routine Expanded Programme on Immunization (EPI), would be expensive and much less cost-effective than direct prevention through PEP ^21^. For post-exposure vaccination, intradermal (ID) administration was under all conditions more cost-effective than the widely used intramuscular route ^21^. An abridged one-week ID regimen was universally preferred over other regimens recognized by WHO as safe and effective, with potential to reduce costs and be more resilient to stockouts during outbreaks ^21^. A dose sparing approach to administration (wound infiltration only) of rabies immunoglobulins was also demonstrated to be more cost-effective than previous recommendations ^21^. These outcomes considerably simplified recommendations for rabies PEP, which had previously been regarded as unwieldy and complex, and informed the latest WHO position ^3^, providing a preferred strategy for improved PEP access under Gavi investment.

Gavi recently announced their support for rabies post-exposure vaccination beginning 2021, subject to funding availability. Models projected that under improved PEP access (Gavi investment), total human rabies vaccine use could remain similar to the status quo across Gavi-eligible countries from 2020–2035 ( ^~^73 million), but with millions more (17.4 million) vaccinated by switching to the abridged one-week ID regimen ^22^. This would prevent around half a million rabies deaths, making improved PEP access an extremely cost-effective intervention at around $600 per death averted and costing approximately $403.7 million for the 46 currently Gavi-eligible countries. The counterfactual prediction was that although current PEP provisioning should save around 900,000 lives from 2021–2035, over one million people will otherwise die from rabies. This difference is attributed to addressing the market failure in vaccine supply, with the introduction of free point-of-care access increasing bite victim care seeking and compliance. Models compellingly showed how investing in PEP would be a step to improve equity and commitment to universal health coverage ^23^. Nonetheless, they also starkly highlighted that without concomitant investment in dog vaccination, the 2030 target of zero dog-mediated rabies deaths cannot be met. Scaling up dog vaccination according to the Zero by 30 strategy is predicted to avert a further 300,000 deaths in addition to those prevented through PEP ^22^. But without dog vaccination, transmission in dogs will become more entrenched and rabies deaths and PEP use are expected to steadily increase in line with population growth.

Vaccinating 70% of dog populations in high-risk areas is a cost-effective and recognized measure to break the rabies transmission cycle ^24, 25^. Empirical work shows that achieving 70% coverage is possible ^26, 27^ and reveals advantages and disadvantages of strategies according to setting ^28– 30^. Models identified heterogeneity in vaccination coverage, and specifically areas of low coverage, as a major impediment to progress ^30, 31^. Coverage gaps can result from limited resources, poor planning, neglect of particular communities, non-participation by local government units and a lack of monitoring and evaluation. Effective education, awareness raising and advertising are key to participation in dog vaccination campaigns and achieving high coverage, as well as for ensuring persons at risk of rabies exposure seek PEP. Models further suggest that rabies persists through metapopulation processes ^32, 33^. Limited geographical extent of dog vaccinations can therefore leave areas vulnerable to incursions from neighbouring endemic populations ^33– 35^. This highlights the need for sustainability, as mass dog vaccination campaigns need maintaining until elimination is achieved in focal areas and their neighbouring populations.

**Figure 1.**
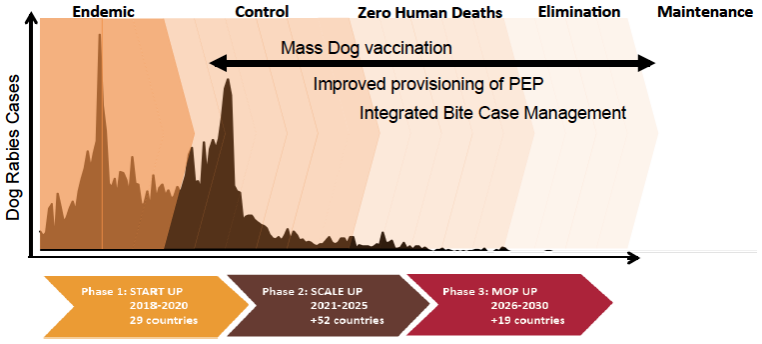
Interventions to reach the Zero by 30 target. PEP, post-exposure prophylaxis.

Another challenge is that even with dog vaccination, PEP costs will likely remain high unless strategies to rationalize PEP are implemented. Integrated Bite Case Management (IBCM) is advocated by WHO, to reduce the costs of PEP once rabies has been controlled ^36^. IBCM is a strategy that formally engages the medical and veterinary sectors to assess the risk of exposure to rabies and requirements for PEP. Implementation of IBCM in Haiti, a resource poor endemic setting, improved patient care by identifying and treating those at risk ^37^, whilst reducing PEP use by 40–60% ^38^. Current PEP access differs dramatically by setting. Chronic shortages occur in the poorest countries where the majority of patients seek care for bites by rabid dogs, whereas access is improved in some middle-income countries and sometimes hundreds of patients are treated for healthy dog bites for every rabies exposure ^39– 41^. In endgame settings, where rabies incidence is very low or absent ( Figure 1), PEP savings could be much higher. Models with conservative implementation of IBCM, i.e. judicious PEP only after countries reach zero human deaths, predict reduced PEP costs by 20–70% ^22^.

## Practical implications of the currently proposed goals

To achieve the targets of the 2030 NTD roadmap, considerable progress must be made in the implementation of rabies control and prevention ( Table 1). PEP must be distributed in sufficient quantities and equitably to all rabies-exposed people ^22^. Gavi support will be crucial and should provide stability and guaranteed funds to maintain production and supply of human vaccines, and catalyse pre-qualification of new suppliers to address ongoing international shortages. In parallel, in most LMICs, mass dog vaccination must be scaled up then maintained at sufficiently high levels through sustained campaigns to interrupt transmission. These efforts should be accompanied by well-planned information campaigns, to ensure that local populations participate and benefit fully from these interventions.

**Table 1.** Summary of challenges for reaching the neglected tropical diseases roadmap targets for rabies.

**Current WHO** **Goal**	Zero human deaths due to dog-mediated rabies by 2030 and milestones of 50% reductions in human rabies mortality and 70% mass dog vaccination coverage in high-risk areas. Regional targets set for interruption of transmission in dogs in the Americas and zero deaths in ASEAN and SAARC countries.
**Technical** **Feasibility**	Technically feasible, but not at current levels of implementation - mass dog vaccination and improved PEP access needs scaling up in most LMICs.
**Requirements**	1. Financial support for dog vaccine procurement and delivery at scale, including large-scale information and awareness raising campaigns 2. Technical assistance for planning, implementation and monitoring of large-scale dog vaccination (e.g. training and supervision, mobile applications, standardized guidance, integrated reporting systems e.g. adaption of DHIS2) 3. Technical assistance for improving PEP access (ID training, DHIS2 adaptation, EPI integration, PEP management and accountability) 4. Targeted approaches for human and animal rabies surveillance (e.g. IBCM, use of RDTs) 5. Novel approaches for dog vaccine delivery in hard-to-reach populations (e.g. ORV, targeted CVR, community-led approaches)
**Challenges**	1. Funding for dog vaccination 2. Limited veterinary capacity 3. Regulatory issues regarding involvement of (lay) non-veterinarian vaccinators 4. Weak surveillance with underreporting of human rabies deaths and animal rabies cases
**Risks**	1. Reliance on PEP to prevent deaths without addressing problem at source, or assessing rabies risk 2. Use of low quality/unregulated animal (and human) vaccines 3. Requirements for private dog vaccination (paid for by dog owners) 4. Use of ineffective methods (culling) and ramifications for public trust and expectations of success 5. Limited technical support for effective surveillance and dog vaccination and its monitoring and evaluation
**Opportunities**	1. Leverage Gavi support for PEP to advocate for dog vaccination 2. Strengthened and harmonized guidance for ‘Zero by 30’ (WHO position paper ^3^, WHO Technical Report Series, OIE endorsement procedures for dog-mediated rabies national control programmes, OIE Terrestrial Code, the rabies blueprint) 3. Pilot projects where catalytic funding has developed in-country capacity and a legacy of ongoing action (Tanzania, Philippines, South Africa, Haiti, Namibia, Bangladesh) 4. Innovative technical assistance (e.g. customized online and onsite training ^43^ - Institut Pasteur, Rabies Action Center of Excellence - FAO) 5. United Against Rabies Coalition (WHO/OIE/FAO/GARC) and committed partners including animal welfare organizations, the pharmaceutical industry, academics, civil society and local rabies champions

WHO, World Health Organization; ASEAN, Association of Southeast Asian Nations; SAARC, South Asian Association for Regional Cooperation; PEP, post-exposure prophylaxis; DHIS2, District Health Information System 2; ID, intradermal; LMIC, low- and middle-income country; EPI, Expanded Programme on Immunization; IBCM, integrated bite case management; RDT, rapid diagnostic test; ORV, oral rabies vaccination; CVR, catch-vaccinate-release; OIE, World Organisation for Animal Health; FAO, Food and Agriculture Organization; GARC, Global Alliance for Rabies Control.

The Zero by 30 strategy ambitiously targets the scale up of dog vaccination in >100 countries over the next decade ^36^. Given the threat of incursions from neighbouring populations, this coordinated approach should accelerate progress. Coordination of dog vaccination across the Americas provides the model for success, but also illustrates how the poorest states impede progress ^42^, i.e. circulation persists where implementation of dog vaccination is weakest, seeding outbreaks elsewhere. Dog vaccination campaigns should be effectively targeted and implemented every year, such that high coverage is maintained locally and nationally, but crucially this requires monitoring and evaluation of campaigns. Moreover, delivery methods need tailoring to local contexts, which may require door-to-door vaccinations ^44^, capture-vaccinate-release ^30^, and possibly supplementary oral vaccination in well-designated areas ^45, 46^, in addition to central point methods ^28^. There is a learning curve to the scaling up of mass dog vaccination. When introduced to communities for the first time, awareness about rabies and trust in dog vaccination may be low, as is the experience and confidence of practitioners, and dog populations estimates may not be very accurate. But over consecutive campaigns dog populations should be better defined and coverage should increase ^47^. This progressive scaling up is recognized by the operational target for countries to achieve 70% vaccination coverage.

Rabies is highly underreported ^16^. Validating the targets of zero deaths and 50% mortality reductions will therefore be a challenge. The most recent WHO expert consultation on rabies included, for the first time, chapters with guidance on validation of zero human deaths and verification of dog-mediated rabies freedom ^2^. The NTD roadmap highlights timely diagnosis and accurate assessment of risks combined with strategic use of rapid diagnostic testing to improve surveillance, i.e. key components of IBCM. Indeed, IBCM is identified as a strategy that can sufficiently enhance surveillance for verification ^48^. Implementation research is, however, urgently needed to provide guidance on operationalization of IBCM across wide-ranging contexts and best practice given anticipated changes over the time horizon until 2030 ( Figure 1). Regulatory measures will be needed for quality control of rapid diagnostic tests and affordable production for larger-scale use.

## Risks

The overwhelming risk to Zero by 30 is the lack of financial support for introduction, implementation and maintenance of mass dog vaccination. Scientific evidence strongly supports the need for sustained high coverage dog vaccination, but most national authorities have not committed sufficient financial resources to support this strategy. Veterinary budgets are typically much smaller than those of health services, and are largely directed towards measures for trade and animal production, not veterinary public health. In most countries that have successfully controlled rabies, the Ministry of Health has led the rabies control programme including financing dog vaccination ^49^. Unless Ministries of Health recognize dog vaccination as an essential public health intervention for human rabies prevention, the Zero by 2030 target will not be achieved. Instead, continued circulation of rabies in dogs may lead to an over-reliance on PEP. International investment in dog vaccine and technical support including training of public health and veterinary personnel ^43^, has demonstrably catalyzed action and is needed to overcome barriers that otherwise prevent rabies activities being initiated and adopted as routine by LMIC governments.

To support effective mass dog vaccination, canine vaccines must meet potency and safety standards ^50^. Use of cheap (substandard) vaccines is a false economy given the much greater costs of vaccine delivery. Furthermore, cheaper, lower quality vaccines can have fatal consequences and dramatically set back elimination programmes ^51, 52^. Countries in Latin America, with long-standing mass dog vaccination programmes and slow progress towards elimination are notably those using vaccines that do not meet international standards ^53^. Some countries push for local vaccine production, but quality standards need to be maintained. World Organisation for Animal Health (OIE) vaccine banks are a step towards more affordable procurement of high quality dog vaccines, but budgets need to be committed to dog vaccination for pharmaceuticals to scale up production, and this remains the most enduring challenge ^54^. Limited regulatory oversight for animal rabies vaccines, which would not be tolerated for human vaccines, is a failure of One Health.

Even in countries with mass dog vaccination, interruptions are common, from financial and logistical challenges to emergencies that divert funds from rabies. Lapses in mass dog vaccination prolong time to elimination ^10, 30^. Rapid dog population turnover causes coverage to decline and recurrent outbreaks can lead to loss of confidence in vaccination ^51^. If dog vaccines are not procured nationally, they may be very limited in availability. Local governments may instead promote culling, which is visible but ineffective, or dog owners may be charged for dog vaccination, leading to ineffectual low coverage ^55^.

A further impediment is that required improvements in surveillance will result in short-term increases in reported cases ^56^. This can have political implications in rabies-endemic countries and lead to resistance to policies that enhance surveillance. Addressing such perverse incentives is a challenge for elimination programmes, where pressure to reach the end can disincentivize reporting. Effective planning and communication is crucial, recognizing the importance of sustainability. Even under strong surveillance, dog vaccination must be maintained for years after cases have ceased to be reported to prevent re-emergence ^57^.

## Future modelling priorities

Modelling now has an important role to play within the 2030 NTDs roadmap, requiring both new and better data, as well as technical development. In anticipation of Gavi investment and with country specific data, modelling should inform where populations are underserved in terms of health care to inform improved provisioning of PEP. Considerable uncertainty in how rabies control and prevention measures will be implemented makes their impacts difficult to ascertain. Forecasting stepwise progress towards goals in the context of data on the roll out of dog vaccination is therefore a priority, with the need for expedient and realistic dynamical models that can be scaled to specific geographies. The size and connectivity of dog populations determines progress towards elimination. Nonspatial models are inadequate for capturing the low endemic incidence of rabies (<1% dogs infected/year); thus, more complex, data-intensive modelling approaches are required. There is scope for such models to prioritize areas for scale up, to predict the duration over which dog vaccinations will be required to eliminate disease and to assess when dog vaccination can be relaxed. Improved data available from the roll out of mass dog vaccinations and post-vaccination monitoring should inform model development and calibration, including dog population estimates and measures of coverage. Improved data collection methods will be invaluable to populating these models. Transition to electronic data collection can provide timely spatially resolved data to better understand effective program implementation ^58^. Programs utilizing mobile applications for large-scale, real-time guidance of vaccination teams has shown effective in both collecting data to improve methodologies and rapidly implementing high-coverage campaigns ^29^. 

Improved dialogue between stakeholders, with a focus on packaging and communicating guidance from models and data, would bring many benefits. Close collaboration between practitioners and modelers to assess the impact and cost-effectiveness of interventions (PEP, dog vaccination, IBCM) could improve implementation, for example, by identifying areas requiring remedial dog vaccination. Models allow the comparison of alternative control strategies, for example, by illustrating the ramifications of low efficacy vaccines or undetected cases from weak surveillance ^57^. Similarly models could alleviate concerns about increased case detection as surveillance improves and rabies awareness builds. Such models would therefore need to include behavioural responses, including to information and awareness campaigns. Burden estimates rely heavily on models. Improved surveillance, particularly IBCM, will provide better data to inform and validate dynamic models and burden predictions, transparently accounting for PEP use and directly measuring impact.

Increasingly, there are calls for integrated interventions in dog populations to more effectively combat diseases that they vector, such as Leishmaniasis, Echinococcosis and Dracunculiasis. Models could help build cost-effectiveness arguments for programmatic integration and inform how such interventions are targeted. Finally, as the goal of elimination approaches, models can be used to compare alternative endgame strategies and guide contingency planning for rapid response and enhanced surveillance to maintain rabies freedom.

## Conclusions

WHO’s 3rd report on NTDs in 2013 highlighted four major obstacles for rabies: improving access to PEP, scaling up of mass dog vaccination, maintaining support for elimination once incidence is no longer a major public health threat, and weak surveillance to monitor progress towards targets ^4^. These challenges remain, but Gavi investment in human rabies vaccines addresses the first, representing an unprecedented and long-overdue recognition of the entirely vaccine-preventable burden of rabies. IBCM can strengthen surveillance over the elimination timeline and prevent PEP costs from escalating, providing a sustainable exit strategy for Gavi and a direct measure of the impact of investment. However, to achieve Zero by 30, Gavi investment needs leveraging to secure country-level and international financing for scaling up mass dog vaccination. Veterinary capacity is limited in most LMICs, and veterinary services have much smaller budgets than Ministries of health. Regulatory mechanisms for animal vaccines are also much weaker than for human vaccines, even though poor quality dog rabies vaccines and poor implementation of dog vaccination results in the deaths of people. The Zero by 30 strategy relies on countries stepping up their dog vaccination programmes over the next five years. The intermediate target of 70% coverage is the critical directly measurable indicator of whether countries deliver on this commitment and investment will be needed to support countries to do so. If countries do not, modelling will remain a counterfactual exercise to demonstrate what could have been achieved and to starkly measure how many deaths from rabies we, the global community, are willing to tolerate.

## Data availability

No data are associated with this article.
