# Supplementary material for: Zero human deaths from dog-mediated rabies by 2030: perspectives from quantitative and mathematical modelling
Source: Gates Open Res. 2020 Mar 4;3:1564. Originally published 2019 Oct 11. [Version 2] doi: 10.12688/gatesopenres.13074.2 (PMC7308633; doi:10.12688/gatesopenres.13074.2)
Supplement: Peer review history

# Peer review: Reviewer response for version 2

**DOI:** 10.21956/gatesopenres.14291.r28843

**Published:** 2020-06-17

**Authors:** Robbie McDonald

**Affiliations:** 1 Environment and Sustainability Institute, University of Exeter, Penryn, UK

In this letter, a consortium of authors describes a pathway towards zero human deaths from dog-mediated rabies, encompassing policy, aid and research, primarily modelling. The case is clear, well articulated and well supported by the literature. The authors highlight the various challenges ahead and identify which of these are likely to be most salient. The letter is also challenging in that it explicitly links the required actions to the models and concludes with the line that if the appropriate action is not taken, then models will serve to highlight how many preventable deaths are tolerable.

 This letter has been reviewed by three other reviewers and the authors have responded constructively to previous reviews. I have no concerns about the letter approach, presentation or conclusions and have no suggestions for further amendments.

Does the article adequately reference differing views and opinions?

Yes

Are all factual statements correct, and are statements and arguments made adequately supported by citations?

Yes

Is the Open Letter written in accessible language?

Yes

Where applicable, are recommendations and next steps explained clearly for others to follow?

Yes

Is the rationale for the Open Letter provided in sufficient detail?

Yes

Reviewer Expertise:

Disease ecology and management. Animal management.

I confirm that I have read this submission and believe that I have an appropriate level of expertise to confirm that it is of an acceptable scientific standard.

# Peer review: Reviewer response for version 2

**DOI:** 10.21956/gatesopenres.14291.r28841

**Published:** 2020-06-16

**Authors:** Michael Z. Levy

**Affiliations:** 1 Department of Biostatistics, Epidemiology and Informatics, Perelman School of Medicine, University of Pennsylvania, Philadelphia, PA, USA

**Overview**

 Overall, the letter, “Zero human deaths from dog-mediated rabies by 2030: perspectives from quantitative and mathematical modelling” is clear and well-written, addressing the contributions modelling studies have made to the ‘Zero dog-mediated human rabies cases by 2030’ initiative as well as the challenging path ahead.

 This letter highlights the evidence provided by modelling studies to clarify and stream line human post-exposure prophylaxis (PEP) recommendations, the considerable impact Gavi investment could have in increasing PEP availability, and how integrated bite case management (IBCM) provides a strategy for rational PEP use.

 The letter also emphasizes that scaling up of canine vaccination efforts are critical to meet the ‘Zero dog-mediated human rabies cases by 2030’ goal. The letter addresses many key challenges including lack of funding of canine rabies vaccinations, underreporting, and more nuanced political challenges. Finally, this letter addresses key areas that modelling studies can help advance progress towards the ‘Zero by 2030’ goal. 

 The letter has already been reviewed thoroughly. We suggest only one additional minor revision.

**Minor comments**

1. Risk section, 1st paragraph, 3rd sentence: 

“Veterinary budgets are typically much smaller than those of health services, and are largely directed towards measures for trade and animal production, not veterinary public health”

 This sentence needs clarification because ‘measures for trade and animal production’ encompass vital veterinary public health measures, such as those necessary for ensuring a safe and secure food supply. Perhaps something along the lines of:

 “Veterinary budgets are typically much smaller than those of health services, and are largely directed towards measures for trade and animal production” would convey the lack of rabies funding without creating a division between trade and production and public health.

Does the article adequately reference differing views and opinions?

Yes

Are all factual statements correct, and are statements and arguments made adequately supported by citations?

Yes

Is the Open Letter written in accessible language?

Yes

Where applicable, are recommendations and next steps explained clearly for others to follow?

Yes

Is the rationale for the Open Letter provided in sufficient detail?

Yes

Reviewer Expertise:

Michael Z. Levy: Disease Ecology, Vector-borne disease control; Brinkley Raynor: Rabies epidemiology, modelling

I confirm that I have read this submission and believe that I have an appropriate level of expertise to confirm that it is of an acceptable scientific standard.

# Peer review: Reviewer response for version 2

**DOI:** 10.21956/gatesopenres.14291.r28649

**Published:** 2020-03-11

**Authors:** Terence P. Scott

**Affiliations:** 1 Global Alliance for Rabies Control, Pretoria, South Africa

I would like to thank the authors again for a very concise, well-constructed and informative manuscript. I have reviewed the revised version and believe that the authors have adequately addressed all of my comments. 

 I noted one last minor spelling inconsistency in Table 1, under "Requirements" point 2. The word 'adaption' is used while in point 3 of the same table, the word 'adaptation' is used. Although both are recognised and accepted words, I would suggest using the same word for consistency's sake.

Does the article adequately reference differing views and opinions?

Yes

Are all factual statements correct, and are statements and arguments made adequately supported by citations?

Yes

Is the Open Letter written in accessible language?

Yes

Where applicable, are recommendations and next steps explained clearly for others to follow?

Partly

Is the rationale for the Open Letter provided in sufficient detail?

Yes

Reviewer Expertise:

Rabies Epidemiology, public health and surveillance

I confirm that I have read this submission and believe that I have an appropriate level of expertise to confirm that it is of an acceptable scientific standard.

# Peer review: Reviewer response for version 1

**DOI:** 10.21956/gatesopenres.14210.r28528

**Published:** 2020-02-13

**Authors:** Terence P. Scott

**Affiliations:** 1 Global Alliance for Rabies Control, Pretoria, South Africa

**Overview:**

 The letter entitled “Zero human deaths from dog-mediated rabies by 2030: insights from modelling”, in my opinion, has been well written and constructed. In brief, the letter summarises the progress that has been made over the last decade in terms of rabies control. Although well documented in published literature, the letter focuses on the role that modelling has played, ranging from the Burden study to advocate for rabies elimination to the various studies relating to the case being made to Gavi for investment in rabies. The letter also highlights many of the challenges faced in terms of achieving the global goal of Zero by 2030, including the need for improved One Health collaboration and improved access and delivery of veterinary vaccines, supplemented with improved PEP delivery, as a core for success. The letter ends with a fantastic concluding sentence as a call for action.

**General comments:**

 Although well-constructed and suitably developed to cover the majority of key issues, the letter fails to address some key factors that would be critical to success in terms of the Zero by 30 goal. These factors would also benefit from modelling studies and thus are directly applicable to the scope of this letter in the sense of knowledge gaps, future needs and addressing the way forward.

 Education is a core factor that has been entirely omitted from the manuscript. Without a solid education foundation, community participation and support will not be possible, thus entirely neutralising any investment in veterinary or human vaccines and vaccination. Furthermore, education of health personnel in terms of the newest WHO recommendations and potential Gavi investment based on the ID regimen will be essential. It would be most helpful if this could be adequately addressed in the letter, including potential means in which modelling could contribute to improving education (through analysing impact).

 The authors have highlighted some extremely important points that truly make the letter a valuable contribution to scientific literature. Some of these points, however, are separated throughout the letter and thus lessen their impact. Upon initial read, the letter seemed to solely pose problems, with few suggested solutions, but upon closer examination the potential solutions or means to address these challenges are in fact mentioned. For example:

- Second paragraph under Future modelling priorities: “Models allow the comparison of alternative control strategies, for example, by illustrating the ramifications of low efficacy vaccines or undetected cases from weak surveillance”. The mention of illustrating the ramifications of undetected cases from weak surveillance through modelling would certainly be critical. However, this point could be strengthened by relating it back to the following sentence: “A further impediment is that required improvements in surveillance will result in short-term increases in reported cases” in the last paragraph of the “Risks” section.

**Minor comments:**

1. Abstract: “Modelling is required for projecting more realistic and geographically specific timelines for achieving targets, in line with the implementation of interventions.” – Are the projection of timelines based on modelling required considering a relatively short time frame to reach the achievable goal of Zero by 30? Based on the content of the rest of the letter, there are other models that should rather be prioritised. This need is reiterated again powerfully in the concluding sentence of the letter.
2. Background, third paragraph: “The Southeast Asia and Western Pacific regions targets for zero deaths from 2020”. Please amend “regions” to “region’s” (possessive).
3. Background, fourth paragraph: “For rabies, the new roadmap, for the first time, identifies time-bound targets for countries…”. Please can you refer to the name of the roadmap for clarity and provide a reference.
4. Figure 1: Please reference this figure
5. Future modelling priorities, first paragraph: “In anticipation of Gavi investment and with country specific data, modelling should inform the spatial targeting of PEP.” This sentence is unclear as to what the goal would be and can be interpreted to be in direct contradiction to the following sentence “PEP must be distributed in sufficient quantities and equitably to all rabies-exposed people” (Practical implications of the currently proposed goals, first paragraph)
6.  Risks, third paragraph: “If dog vaccines are considered expensive, governments may instead promote culling…”. Are dog vaccines themselves considered expensive in rabies endemic countries, or is this rather due to the cost of delivery? Furthermore, the related logistical efforts needed (typically due to poor planning) for dog vaccination often make culling seem a more appropriate and feasible short-term answer. I suggest rephrasing to “If effort and cost related to dog vaccination seem excessive, governments…”
7. Conclusions: “Veterinary capacity is limited in most LMICs, with small budgets compared to health, which are not investing in veterinary public health despite the ‘One Health’ rhetoric.” – this sentence is ambiguous. Please rephrase.
8. Conclusions: “The intermediate  target of 70% coverage is the critical directly measurable indicator” – Although 70% coverage is a directly measurable indicator, it remains challenging to measure based on the lack of data or estimates for dog populations in different geographic locations, especially considering both owned and unowned/free-roaming dogs should be included. Including a more accurate and feasible means to estimating dog populations in future modelling priorities would certainly be something worth consideration and discussion.   
9. References: Reference 14 is incomplete.

Does the article adequately reference differing views and opinions?

Yes

Are all factual statements correct, and are statements and arguments made adequately supported by citations?

Yes

Is the Open Letter written in accessible language?

Yes

Where applicable, are recommendations and next steps explained clearly for others to follow?

Partly

Is the rationale for the Open Letter provided in sufficient detail?

Yes

Reviewer Expertise:

Rabies Epidemiology, public health and surveillance

I confirm that I have read this submission and believe that I have an appropriate level of expertise to confirm that it is of an acceptable scientific standard, however I have significant reservations, as outlined above.

# Response

**Published:** 2020-02-24

**Authors:** Katie Hampson

*We are grateful for the positive review. We agree entirely with the point on the importance of education and awareness raising, which was also highlighted by the first reviewer. We have edited the manuscript to emphasise this need and indicate how modelling and analysis could be used to measure the impact of such efforts.*

*We are also grateful for the reviewer pointing out some slightly disjointed points in our manuscript.  We have tried to better indicate potential solutions to the challenges that we raise, and have restructured in places or directed the reader to where solutions are discussed.*

*We have also tried to address each of the minor points raised by the reviewer, including revising the text to improve clarity (in the abstract, the Future modelling priorities section, the risks section and conclusion), correcting punctuation, referring to figures clearly, correcting references and citing as indicated.*

# Peer review: Reviewer response for version 1

**DOI:** 10.21956/gatesopenres.14210.r28107

**Published:** 2019-11-27

**Authors:** Florence Cliquet

**Affiliations:** 1 Nancy Laboratory for Rabies and Wildlife, French Agency for Food, Environmental and Occupational Health & Safety (ANSES), Malzéville, France

The paper Zero human deaths from dog-mediated rabies by 2030: insights from modelling is a short review of the global state of the art of the Zero by 30 strategy. The paper is well constructed, detailing the background of the initiative, the main challenges and the global results achieved so far. The priorities for future modelling are described.

 The title of the manuscript is not completely appropriate, as most information given in the paper is not linked to the modelling approach. All the challenges and the problems described have been identified since a long time and will not be solved by a modelling approach.

 In this quite recent initiative, the veterinary public authorities are in charge of the most difficult task, i.e. mass vaccination of dogs, mainly because of a lack of organisation and infrastructure in many countries because rabies and dogs are not a political priority. Vaccinating every year 70% of the dog population, even with money, is highly difficult to implement in a so short time (in Americas, it required more than 30 years), as it mainly requires planning, coordination, organisation and evaluation of the implemented actions during all the year and in local settings. The methodology with the different techniques to reach dogs as well as gaps are known and published in many papers; It needs also sustainability for a long time (this notion of sustainability should be mentioned in the paragraph starting by Vaccinating 70% of dog….). So the paper is too severe when concluding on the actions undertaken by the veterinary authorities that are judged as insufficient (….veterinary services focused on …..). The paper should more reflect the difficulty of this task and should also insist on the fact that the One Health approach should be elaborated concretely at the local basis, even on limited areas at the start, associating the people of MoH and MoA to work together, at the same time, particularly for IBCM. The One Health approach is precisely aimed to create concrete collaborations within the different stakeholders to enhance control and prevention.

 The notions of education and awareness of the population are not sufficiently stressed in the paper, they are essential. Rabies surveillance and control are more efficient when the population participates closely with the veterinary services, particularly for reporting suspect cases (surveillance) and for presenting animals during mass parenteral vaccination campaigns. The paper of Ripani (Ripani A., Mérot J., Bouguedour R., Zrelli M. Review of rabies situation and control in the North African Region with a focus on Tunisia ^1^) should be added to the reference list, showing clearly the importance of awareness of the population.

 The sentence Dog vaccination campaigns must be…. should be changed for Dog vaccination campaigns should be effectively targeted and implemented every year, such that high coverage……”.

 OVD is an expensive method rhetorically miraculous, however the drawbacks are numerous as soon as we test it in the field in local conditions; this method is to be used for countries close to elimination, which are already engaged in efficient mass parenteral vaccination with good results, and which have still rabies pockets in defined areas. Therefore, the following sentence has to be moderated for: “Moreover, delivery methods need tailoring to local contexts, which may require door-to-door and central point method vaccinations, capture-vaccinate-release, and possibly supplementary oral vaccination in well designed areas. Please insert in this sentence a recent review on OVD (Cliquet F., Guiot A.L., Aubert M., Robardet E., Rupprecht C.E., Meslin F.X. - Oral vaccination of dogs: a well-studied and undervalued tool for achieving human and dog rabies elimination ^2^).

 The reference 44 is not cited in the text.

 A reference (Taylor E., Banyard A.C., Bourhy H., Cliquet F., Ertl H., Fehlner-Gardiner C., Horton D.L., Mani R.S., Müller T., Rupprecht C.E., Schnell M.J., Del Rio Vilas V., Fooks A.R. - Avoiding preventable deaths: The scourge of counterfeit rabies vaccines ^3^.) should be added for the sentence “Use of cheap (substandard) vaccines is a false……”.

Does the article adequately reference differing views and opinions?

Yes

Are all factual statements correct, and are statements and arguments made adequately supported by citations?

Partly

Is the Open Letter written in accessible language?

Yes

Where applicable, are recommendations and next steps explained clearly for others to follow?

Partly

Is the rationale for the Open Letter provided in sufficient detail?

Yes

Reviewer Expertise:

Rabies immunology, vaccinology and epidemiology

I confirm that I have read this submission and believe that I have an appropriate level of expertise to confirm that it is of an acceptable scientific standard, however I have significant reservations, as outlined above.

## References

[1] : Review of rabies situation and control in the North African region with a focus on Tunisia. *Rev Sci Tech*.2017;36(3) : 10.20506/rst.36.3.2718 831-838 10.20506/rst.36.3.2718 30160705

[2] : Oral vaccination of dogs: a well-studied and undervalued tool for achieving human and dog rabies elimination. *Veterinary Research*.2018;49(1) : 10.1186/s13567-018-0554-6 10.1186/s13567-018-0554-6 30005701PMC6045873

[3] : Avoiding preventable deaths: The scourge of counterfeit rabies vaccines. *Vaccine*.2019;37(17) : 10.1016/j.vaccine.2019.03.037 2285-2287 10.1016/j.vaccine.2019.03.037 30922698

# Response

**Published:** 2020-02-24

**Authors:** Katie Hampson

*We are grateful for the positive review and understand the comment that many of the problems raised in the manuscript will not be solved by a modelling approach. Our intention was to highlight those issues that make modelling challenging rather than indicating that modelling would solve them. In our revision we have tried to be clearer on this.*

*We have highlighted the need for sustainability in the paragraph suggested and have revised the text to indicate the importance of One Health and intersectoral collaboration to achieve these ambitious goals.*

*We are grateful for the reviewer highlighting the three highly relevant references, which we now include together with appropriate edits to reflect the reviewer’s suggestions.*

*We also corrected the sentence on dog vaccination as suggested. We note that reference 44 is cited in Table 1. We also now cite the reference in the main text.*
